# Regulatory T Cells in Radiotherapeutic Responses

**DOI:** 10.3389/fonc.2012.00090

**Published:** 2012-08-17

**Authors:** Dörthe Schaue, Michael W. Xie, Josephine A. Ratikan, William H. McBride

**Affiliations:** ^1^Division of Molecular and Cellular Oncology, Department of Radiation Oncology, David Geffen School of Medicine, University of California at Los AngelesLos Angeles, CA, USA

**Keywords:** radiation, danger, Tregs

## Abstract

Radiation therapy (RT) can extend its influence in cancer therapy beyond what can be attributed to in-field cytotoxicity by modulating the immune system. While complex, these systemic effects can help tip the therapeutic balance in favor of treatment success or failure. Engagement of the immune system is generally through recognition of damage-associated molecules expressed or released as a result of tumor and normal tissue radiation damage. This system has evolved to discriminate pathological from physiological forms of cell death by signaling “danger.” The multiple mechanisms that can be evoked include a shift toward a pro-inflammatory, pro-oxidant microenvironment that can promote maturation of dendritic cells and, in cancer treatment, the development of effector T cell responses to tumor-associated antigens. Control over these processes is exerted by regulatory T cells (Tregs), suppressor macrophages, and immunosuppressive cytokines that act in consort to maintain tolerance to self, limit tissue damage, and re-establish tissue homeostasis. Unfortunately, by the time RT for cancer is initiated the tumor-host relationship has already been sculpted in favor of tumor growth and against immune-mediated mechanisms for tumor regression. Reversing this situation is a major challenge. However, recent data show that removal of Tregs can tip the balance in favor of the generation of radiation-induced anti-tumor immunity. The clinical challenge is to do so without excessive depletion that might precipitate serious autoimmune reactions and increase the likelihood of normal tissue complications. The selective modulation of Treg biology to maintain immune tolerance and control of normal tissue damage, while releasing the “brakes” on anti-tumor immune responses, is a worthy aim with promise for enhancing the therapeutic benefit of RT for cancer.

## Radiation and “Danger” Signaling

Local RT has complex, systemic consequences (Formenti and Demaria, 2009) that, if harnessed properly have the power to significantly shape host-tumor relationships and ultimately affect treatment outcome. This review will focus on those aspects of RT that could translate into anti-tumor immunity, and their immune regulation.

Tissues that have been damaged by radiation display various “danger” signals to the immune system that can be secreted and/or released into extracellular spaces. The so-called Damage-Associated Molecular Pattern molecules (DAMPs; Shi et al., 2003; Lotze et al., 2007; Curtin et al., 2009; Sato et al., 2009). What characterizes DAMPs is that they are endogenous molecules that signal through a set of common pattern recognition receptors (PRRs; Matzinger, 2002; Lotze et al., 2007; Kawai and Akira, 2011), such as the Toll-like receptor (TLR) family (Medzhitov et al., 1997; Beutler, 2009), nucleotide binding oligomerization domain (NOD)-like, and retinoic acid inducible gene (Rig)-like receptors (Meylan et al., 2006), and C-type lectins (Robinson et al., 2006). Once engaged, PPRs initiate signaling cascades to establish communications between immune cells through generally pro-inflammatory cytokine and chemokine networks. The system has evolved to recognize and deal with dangerous pathological situations, restore homeostasis, and to regenerate and heal tissues (Schaue and McBride, 2010; Schaue et al., in press).

Within tumors, DAMPs are generated by cell stress and death during progressive growth and increasing vascular abnormalities, and by oxidative damage and hypoxia (Ullrich et al., 2008; Sato et al., 2009). DAMP signaling and the cytokines they generate not only affect the content and function of innate immune cells within tumors, but also can play critical roles in the generation of adaptive immunity. This is because dendritic cells (DCs) have to mature to be competent at antigen-presentation, which requires pro-inflammatory “danger” signals (Banchereau and Steinman, 1998; Gallucci et al., 1999). Mature DCs are crucial for providing signal 2, the verification co-stimulatory signal that is needed to translate signal 1 (antigen) into a T cell-mediated immune response. Conversely, antigen-presentation in the absence of co-accessory signaling leads to immune tolerance (Steinman et al., 2003). In cancer treatment, the potential role of DAMP recognition and the initiation of adaptive anti-tumor immunity is seen in breast cancer patients with defective TLR-4 signaling who are less able to respond to standard therapy presumably because of a lack in tumor immune eradication (Apetoh et al., 2007). There is however a possible negative side to this equation as all cells, including tumor cells, express DAMP receptors of varying types which can drive tumor progression (Sato et al., 2009).

Tumor RT certainly will increase the amount of DAMPs released, but the extent to which it qualitatively and quantitatively changes DAMPs levels is not known, nor how such changes will affect the immune responses that are made. Exacerbation of the level of “danger” signaling in the tumor microenvironment by RT has however the potential to activate innate immune cells and link to the development of tumor antigen-specific, adaptive immunity. In support, we, and others, have observed that radiation can mature DCs, enhancing expression of numerous molecules that further aid immune recognition, such as MHC class I and II molecules, co-stimulatory CD80, cell adhesion molecules such as ICAM-1, integrins, and selectins, and damage recognition molecules such as phosphatidyl serine (Santin et al., 1996; Morel et al., 1998; Seo et al., 1999; Garnett et al., 2004; Reits et al., 2006; Tyurina et al., 2011), in addition to creating a pro-oxidant, pro-inflammatory milieu that encourages infiltration by immune cells (Lorimore et al., 2001; Lugade et al., 2005, 2008; Matsumura et al., 2008; Burnette et al., 2011). Overall, these responses seem to be a deliberate attempt by the tissue to improve immune cell access and to encourage immunogenicity and susceptibility to attack by T lymphocytes and other immune cells (Garnett et al., 2004). For example, irradiated tumor cells can show enhanced expression of the death receptor Fas *in vitro* and *in vivo*, consequently sensitizing tumors to antigen-specific cytotoxic T cells and, ultimately, rejection (Chakraborty et al., 2003, 2004).

A case can therefore be made for cancer therapies like RT being able to act as immune adjuvants, in addition to having direct anti-tumor action (Roses et al., 2008). Such responses must be carefully controlled. Optimization of anti-tumor immune responses following RT is not trivial and requires consideration of many additional contributing factors.

## Radiation as an Immune Adjuvant

If RT can induce a pro-oxidant, pro-inflammatory microenvironment, one would expect that irradiated tumors often induce measurable systemic immune responses that can lead to tumor regression in preclinical models (Lugade et al., 2005; Lee et al., 2009; Perez et al., 2009; Spanos et al., 2009). There are a few encouraging reports indicating that humans receiving RT may make increased immune responses when combined with other immunostimulatory therapies (Nesslinger et al., 2007; Ferrara et al., 2009; Stamell et al., 2012), with chemotherapy or even alone (Schaue et al., 2008). In the last example, we showed that circulating tumor-specific CD8+ T cells can rise in colorectal cancer patients toward completion of chemo-radiation with 45 Gy and continuous 5-fluorouracil infusion (Debucquoy et al., 2006, 2009; Schaue et al., 2008). More general support for the view that the immune system can be a powerful and independent prognostic indicator of a good response to cancer therapies comes from studies on T cells infiltration in solid tumors (Galon et al., 2006; Pages et al., 2010) and from abscopal effects that can be attributed to the systemic development of immunity (Formenti and Demaria, 2009; Stamell et al., 2012). Questions however remain as to why tumor-specific responses are not always generated by therapies, even within one tumor type, why some types of tumors generate such responses only rarely, and the ultimate question of why tumors continue to grow even in the presence of an immune response that appears effective *in vitro*.

One issue that must be considered is that by the time therapy is initiated tumors have already escaped the attentions of the immune system. Multiple mechanisms have been described by which this is achieved (Zitvogel et al., 2006; Whiteside, 2009). The nature of the immune escape mechanism strongly influences the tumor-host relationship, the tumor antigens that are expressed, and probably the outcome of any therapeutic approach. For example, even highly immunogenic tumors can grow progressively and maintain strong tumor antigen expression if they generate powerful suppressor T cells and macrophages (Howie and McBride, 1982; McBride and Howie, 1986; Iwai et al., 2002). On the other hand, tumors may undergo immunoediting that selects for cells lacking antigen expression during tumor development. In the former situation, tumors are more likely to respond to removal of immune suppressor cells than in the latter. In some tumors, the rate of tumor cell death and turnover could be critical in balancing the immune system so as to favor tumor growth. In this case, simply changing this equation through aggressive therapies may have a positive effect. In each of these scenarios, the tumor antigens that are expressed are likely to differ in potency for stimulating immunity and the suppressor mechanisms that have to be overcome will vary in strength and type. This indicates that different strategies for potentiating tumor immunity may need to be tailored to the existing state of the tumor-host relationship. Additional factors that might limit the generation of the “dangerous” microenvironment and the extent of adaptive immunity to the tumor include the nature of the vasculature, the degree of oxidative stress, and the extent of hypoxia in a tumor (Conejo-Garcia et al., 2004; Rius et al., 2008; Sitkovsky, 2009; Facciabene et al., 2011; Kandalaft et al., 2011). RT has been shown to change the tumor microenvironment by causing vascular damage, inhibiting angiogenesis, and enhancing chronic hypoxia at the expense of transient hypoxia, with the newly generated hypoxic areas becoming infiltrated with tumor-promoting macrophages (Dewhirst et al., 1990; Garcia-Barros et al., 2003; Chen et al., 2009; Ahn et al., 2010; Kioi et al., 2010). These crucial variables may shape the tumor response to RT and vary with the tumor and its location (Chiang et al., 2012).

The dose and delivery schedule for RT also influences the development of anti-tumor immunity. For RT to be an immune adjuvant there seems to be an optimal size of dose and dose per fraction, with moderate dose fractions of around 5–6 Gy being superior to 2 Gy fractions (Dewan et al., 2009; Schaue et al., 2012). And in the case of the murine melanoma model, tumor-specific immune responses following RT were found to inversely correlate with tumor size illustrating an interesting dichotomy in the tumor-host relationship (Schaue et al., 2012). These findings generally support the belief that therapy-induced tumor damage can translate into measurable immune activation.

## Limiting the Immune Response to Protect Self

The transition from the rapidly generated, innate immune response to activation of the slower, more sophisticated adaptive immune system is a critical step in the development of tumor immunity. Importantly, adaptive immunity tends to be polarized, especially with respect to antigen-specific helper and regulatory T cell subsets (Th/Tregs; Fernandez-Botran et al., 1988) that can ultimately dictate immune-mediated regression or progression, most often mediated through CD8^+^ T cell activation. CD4^+^ naïve cells (Th0) recognize antigenic peptides on DCs through their T cell receptor-CD3^+^ complexes and, based on the signals received, can differentiate along one of at least four pathways to form Th1, Th2, Th17, or iTregs. This dramatic cellular polarization is orchestrated by the prevailing cellular microenvironment through a network of transcription factors and microRNAs; T bet for Th1, GATA-3 for Th2, RORgammat for Th17 cells, and Foxp3, miR-10a, miR-155 for Tregs (Zhu and Paul, 2010; Dang et al., 2011; Gao et al., 2012; Takahashi et al., 2012).

The important result is the emergence of T cell subsets that, while they are antigen-specific, exert much of their influence through distinctive effector cytokine profiles that influence bystander non-immune and immune cells alike, depending upon their cytokine receptor patterns. Th1 cells respond primarily to IL-12 to produce IFN-γ, GM-CSF, and TNF-α and are important for assisting cytotoxic CD8^+^ T cell-mediated responses that can eliminate tumors. They also activate macrophages to express a pro-inflammatory phenotype that can be cytotoxic to tumors. Th2 cells, in contrast, are stimulated primarily by IL-4 to produce IL-4, IL-5, IL-6, IL-13, and IL-25. They assist B cells in the generation of antibodies that form allergic responses. Th17 cells differentiate in response to IL-6 or IL-22 to produce IL-17, IL-21, IL-22, IL-23, and GM-CSF. Th17 cells have been implicated in the pathogenesis of many chronic inflammatory and autoimmune diseases (Waite and Skokos, 2012). The concept that distinct functional T cell subsets exist as balanced forces to maintain homeostasis has established validity and has been extended to CD8^+^ T cells, “classically” activated M1, and “alternatively” activated M2 macrophages and DC1/DC2 DCs (Czerniecki et al., 2001; Van Ginderachter et al., 2006), although there is some controversy as to the degree of reprogramming that is possible within these other immune cell types.

As crucial for tumor immunity and as life-saving as any of the above immune players are, the mutual antagonism that exists between different Th subsets in itself is insufficient to control the immune system, which can cause extensive tissue damage if left unrestrained, as in chronic inflammation, autoimmune, and allergic reactions. Tregs (also known as suppressor T cells) are the major players in preventing excessive damage to self (Peterson, 2012) and they represent that other side of the immunological coin from Th cells. The presence of T cells that could suppress antigen-specific inflammatory T cell activity was first recognized by Gershon and Kondo (1971), who called the phenomenon “infectious immunological tolerance.” Plagued by lack of appropriate markers for T cell subpopulations, the Treg field fell into disrepute for many years, but re-emerged with the discovery of Tregs that are now known to fall into two major subsets of natural (nTregs) and induced (iTregs). These have largely non-overlapping distinct antigen recognition repertoires (Haribhai et al., 2009, 2011). Unlike Th cells, both Treg subsets focus on recognition of “self” antigens to maintain peripheral immunological tolerance and exert homeostatic control over inflammation through release of immunosuppressive cytokines (Bluestone and Abbas, 2003; Curotto de Lafaille and Lafaille, 2009).

## Tregs Make Us Tolerant of Our Self and of Others

The importance of Tregs in maintaining peripheral self-tolerance, preventing autoimmune disease, and limiting inflammation and immunity (Sakaguchi, 2004; Shevach, 2004) is exemplified by the havoc caused in their absence, ranging from excessive lymphoproliferation, immune, and inflammatory tissue damage, to death. For example, a loss-of-function mutation in the essential regulator of Tregs, the forkhead box transcription factor Foxp3, leads to a lethal autoimmune and inflammatory disorder in the “scurfy” mouse and the IPEX syndrome (Immune dysregulation Polyendocrinopathy Enteropathy X-linked Syndrome) in humans (Fontenot and Rudensky, 2005; Chatila, 2009). Interesting in this context is the fact that high fractionated doses of radiation delivered to the lymphoid system of mice also generates autoimmunity (Sakaguchi et al., 1994).

Tregs function in widely diverse scenarios to control other T and B lymphocyte subsets, DCs, and macrophages, as well as non-immune cells. Although T cell receptor recognition and activation is through cognate antigen, suppression in their immediate environment can be rather indiscriminate, at least *in vitro* (Shevach, 2009). They use various immunosuppressive effector mechanisms, any one of which may be favored under specific conditions (Pillai et al., 2011). These include cell-to-cell contact, the release of cytokines such as IL-10, IL-4, IL-35, and/or TGF-β, and the production of adenosine that drives cAMP elevation and inhibition of T effector cells (Chen et al., 2005; von Boehmer, 2005; Deaglio et al., 2007; Shevach, 2009; Efimova et al., 2011). By generating an anti-oxidant/adenosinergic microenvironment, Tregs are tissue protective and the antithesis of pro-oxidant acute inflammation.

Most Tregs are naturally occurring, functionally mature CD4^+^CD25^hi^Foxp3^+^ Tregs (nTregs) that are “hard-wired” with respect to their immune repertoire through thymic development and are already primed for suppressive function. In contrast, CD4^+^CD25 ^−^ naïve T cells can be converted outside the thymus into CD4^+^CD25^hi^Foxp3^+^ Tregs, and are therefore called inducible or adaptive, iTregs. Induction can be a result of exposure to low doses of antigen, IL-2, and TGF-β (Apostolou and von Boehmer, 2004; Curotto de Lafaille et al., 2004). Given these differences in origin, it is not surprising that recombinase-deficient mice can generate iTregs but have no nTregs (Curotto de Lafaille et al., 2001; Mucida et al., 2005).

**Figure 1:**
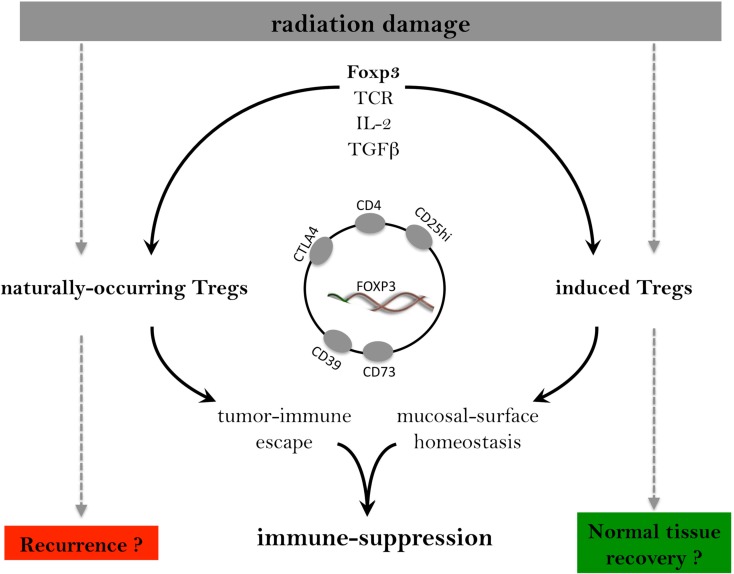
**Systemic immune control is exerted through the combined effort of thymically derived, naturally occurring nTregs, and peripherally induced iTregs that have specificity for “self” antigens but with distinct, minimally overlapping TCR repertoires**. Both Treg pools depend heavily on the transcription factor FoxP3 and on IL-2 with TGF-β providing additional stimulation. While both Treg subsets contribute to immune suppression, iTregs seem to be selectively involved in mucosal surfaces. Radiation therapy drives an increase in Tregs that may limit potential anti-tumor immunity and aid tumor escape on one side but that may also nurture normal tissue recovery on the other.

The functional distinction between iTregs and nTregs has still to be fully established, but they do not share the same workload in controlling the adaptive immune response. Overall, the regulatory phenotype of iTregs and their Foxp3 expression is less stable than that of nTregs possibly due to differences in epigenetic regulation and microRNA miR-10a availability (Floess et al., 2007; Takahashi et al., 2012). Their gene expression profiles are not identical (Feuerer et al., 2010). Molecular studies indicate that nTregs, but not iTregs, express Helios, an Ikaros family transcription factor (Thornton et al., 2010) and are activated by TNF-α (Housley et al., 2011) and by IL-6, the latter converting them to Th17 cells that can mediate potentially pathogenic autoimmunity (Xu et al., 2007). iTregs resist such Th17 conversion (Zheng et al., 2008). These differences may be important in that there is some evidence that iTregs exert control of inflammatory responses at normal mucosal surfaces while nTregs appear more important for mediating self-tolerance and tumor immune escape (Sakaguchi, 2004, 2005; Curotto de Lafaille and Lafaille, 2009; Haribhai et al., 2011; Rosenblum et al., 2011; Josefowicz et al., 2012; Figure 1). There is a distinct possibility that RT might differentially affect these Treg subpopulations, but this has yet to be established.

## Radiation Effects on Immunity *in vivo*

The concept that RT is purely immunosuppressive because lymphocytes are very radiation sensitive is out-moded. While scientific wisdom indicates that lymphocytes are very radiosensitive, subsets differ in this regard and because all immune cells can be induced by radiation itself, as well as by DAMPs, cytokines, and other stimuli to respond at the molecular level, RT is clearly better regarded as being immunomodulatory. In very general terms, a spectrum of radiosensitivity exists from B cells through naive Th cells, NK cells, T memory cells (Belka et al., 1999), Tregs, and DCs to radioresistant macrophages, with a tendency toward apoptosis denoting a more radiosensitive phenotype and non-proliferative cells and activated lymphocytes being more radioresistant (McBride et al., 2004). As a result of blood flow through the field, even local RT will have a purely physical cytotoxic effect of the circulating immunocyte pool, which will vary with the tissue, and the delivery time and dose. Induced responses in tumor and normal tissues, and in the immune cells themselves add considerable additional complexity to the immune equation. The usual radiobiological parameters such as dose, dose rate, fraction size, and radiation quality are pertinent in all cases. Further, if chemotherapy is also given, different drugs are expected to target different immune cell populations, again with dose and scheduling being important parameters.

The ability of radiation to differentially modulate T cell subsets was in fact observed by North, Hellstroem, and others more than 30 years ago. They showed that sublethal, whole-body irradiation eliminated suppressor T cells leading to partial or complete tumor regression in immuno-competent, but not in immuno-incompetent, mice (Hellstrom et al., 1978; Tilkin et al., 1981; North, 1986). The same subset appeared sensitive to low dose cyclophosphamide (Bonavida et al., 1979; Awwad and North, 1989). This introduced the concept of metronomic low dose chemotherapy treatment that might assist elimination of immune suppressor cells, but angiogenesis and other cells are also possible targets (Penel et al., 2012). In contrast to these studies, we and others have shown that Tregs are relatively radioresistant (Kusunoki et al., 2010; Nakatsukasa et al., 2010; Qu et al., 2010; Weng et al., 2010; Kachikwu et al., 2011). A possible explanation for this discrepancy lies in the fact that the timing of the radiation exposure post-tumor implantation was critical in North’s experiments and that a Treg subpopulation may have been induced that became sensitive to radiation. Although Tregs have often been considered inherently anergic, robust Treg proliferation has been observed after stimulation (Walker, 2004). The sensitivity of Tregs to chemo- and radiotherapy in cancer patients is of great clinical interest but largely unknown. The suggestion is that there are immune mechanisms of action as an alternative to direct cytotoxicity, although at present there are no definitive data. In fact, there may be other immune targets such as the myeloid cells that can be induced following RT and whose elimination enhances radiation-induced tumor regression (Ahn et al., 2010).

What we do know is that the tumor-specific immune responses made by cancer patients receiving RT appear to be held in check by increases in the systemic Treg pool (Schaue et al., 2008). We have seen this phenomenon also in murine tumor models mice treated with radiation (Schaue et al., 2012). Interestingly, radiation can increase Treg representation even in the absence of a tumor (Cao et al., 2009; Kusunoki et al., 2010; Nakatsukasa et al., 2010; Qu et al., 2010; Billiard et al., 2011; Kachikwu et al., 2011). This can be interpreted as a response to control radiation-induced inflammation and normal tissue damage. One possible mechanism is through induction and activation of the powerful immune-suppressive cytokine TGF-β by RT (Martin et al., 2000), which is known to boost Tregs (Chen et al., 2003; Beal et al., 2012; Takahashi et al., 2012). In addition, we were able to detect radiation-enhanced expression of the ectonucleotidase CD39 on the Treg population, which has also been observed in treated cancer patients (Mandapathil et al., 2009). Adenosine production through nucleotide catabolism by CD39 and CD73 is probably the most primitive immunosuppressive response to “danger.” Adenosine has long been known to play a critical, non-redundant role in the protection of normal tissues from collateral damage during inflammation (Cronstein, 1994), including radiation-induced tissue damage (Hosek et al., 1992;Pospisil et al., 1993, 1998; Hou et al., 2007), where it plays a protective role (Hofer et al., 2002). Support for this scenario comes from the observation that tissue derived adenosine acting through its receptor A_2A_R drives Tregs and limits autoimmune tissue destruction (Zarek et al., 2008).

## Inhibit the Inhibitors to Widen the Radiotherapeutic Window?

The existence of tumor-induced immunosuppressive T cells and myeloid cells has been known for decades (Howie and McBride, 1982) and Tregs may influence the development of suppressor macrophages through cytokine release. It has taken longer for the concept that the immune system is under continuous negative regulation to be recognized and that loss of these important control mechanisms under steady state conditions can augment inflammation and autoimmunity. Importantly, tools are now available for investigating the role of these subsets in RT settings and for modifying their influence.

There are numerous reports that myeloid-derived suppressor cells (MDSC) and Treg levels are elevated in the peripheral circulation of cancer patients. They are also increased in lymphoid organs and tumors of tumor-bearing mice (Howie and McBride, 1982; Chen et al., 2009). Further, systemic depletion of Foxp3^+^ Tregs enhances natural as well as vaccine-induced anti-tumor T cell responses (Liyanage et al., 2002; Curiel et al., 2004; Dannull et al., 2005; Miller et al., 2006), as does targeting CD11b^+^ myeloid cells (Ahn et al., 2010). It is now generally accepted that a rise in MDSC or Tregs in a patient’s blood or tumor is often associated with poor outcome and that this can be attributed to their immunosuppressive and/or tumor growth promoting effects. The possible exceptions are colorectal and head and neck cancers (Ladoire et al., 2011; Deleeuw et al., 2012), which may indicate greater microbial involvement in these sites. Also, it is difficult to reliably conclude that a rise in Tregs is a negative prognostic indicator if simultaneous measurements are not made in cytotoxic immune cells, the reason being that any pro-inflammatory response is likely to solicit an adaptive compensatory response (Litjens et al., 2012; Tang et al., 2012). In this sense, Tregs may be considered as another immunological readout that mirrors the development of cytotoxic effector T cells, further supporting the general thesis that radiation can be an immune adjuvant (Schaue et al., 2008). Both Tregs and myeloid suppressor cells may be viewed as wound healing responses to tissue damage, only in this case the damage is caused by tumor growth.

From an immunological perspective, the challenge for cancer RT is to create an immunologically permissive environment. This is complex with many pre-existing and induced negative regulatory barriers to be overcome. The size of the challenge will vary with the pre-existing tumor-host environment, the clinical stage and type of tumor, the condition of the patient, and many other variables. These hurdles will vary in height and it may not be possible to generate observable responses in all cases. However, some approaches to unmasking the adjuvanticity of RT show considerable promise.

One of the most effective ways to overcome such barriers is through broad Treg targeting with anti-CD25 antibody and/or immunotoxin or anti-CTLA-4 antibody (Leach et al., 1996; Rasku et al., 2008; Hodi et al., 2010; Byrne et al., 2011; Mellman et al., 2011). Enhanced anti-tumor immunity in general and the effectiveness of RT in particular have been shown (Demaria et al., 2005; Kachikwu et al., 2011; Postow et al., 2012). Currently, the extent of any Treg subset selectivity in these approaches is not known, nor whether radiation-induced normal tissue complications are increased. The use of anti-CTLA-4 as a monotherapy (Phan et al., 2003; O’Day et al., 2007; Yang et al., 2007; Weber et al., 2009), for example, is associated with some toxicity and should be used with caution when combined with other therapies. Furthermore, there are suggestions that Foxp3 may not always be a desirable target in every cancer setting because Foxp3^+^ T cell infiltration does not always predict poor prognosis, for example in colorectal cancer, and because Foxp3 appears to act as a tumor suppressor gene when expressed in non-immune tissues (Deleeuw et al., 2012; McInnes et al., 2012). The influence of myeloid cells may be decreased by colony stimulating pathways on which they depend (Ahn et al., 2010; Vincent et al., 2010), but once RT or chemotherapy is over, both are likely to rebound, which may be the best time to target these brakes on the development of anti-tumor immunity. The potential power of these immunological approaches is very appealing and they may be enhanced even more in the future by more selective targeting of tumor-specific Treg TCRs with antibodies to eliminate those driving immune suppression or with cytokines that could enhance macrophage anti-tumor action or drive Tregs into an effector mode (Byrne et al., 2011).

## Conflict of Interest Statement

The authors declare that the research was conducted in the absence of any commercial or financial relationships that could be construed as a potential conflict of interest.
